# An acute bleeding metastatic spinal tumor from HCC causes an acute onset of cauda equina syndrome

**DOI:** 10.7603/s40681-015-0018-5

**Published:** 2015-08-23

**Authors:** Chih-Ying Wu, Hsiang-Ming Huang, Der-Yang Cho

**Affiliations:** Department of Neurosurgery, China Medical University Hospital, 404 Taichung, Taiwan

**Keywords:** Cauda equina syndrome, HCC, Metastatic spinal tumor

## Abstract

Hepatocellular carcinoma (HCC) is an aggressive tumor that frequently occurs in the setting of chronic liver disease and cirrhosis. Herein, we describe a case where a patient presented with acute onset cauda equina syndrome due to an intradural and extramedullary metastatic tumor bleeding from hepatocellular carcinoma (HCC). The patient had lower back pain that had radiated to the bilateral lower legs for 3 weeks. Then, the patient had experienced an acute onset of bilateral lower leg weakness as well as bladder-urinary dysfunction 2 days before going to the ER. The patient received a laminectomy from the L1 to L4 vertebra, removing the intradural spinal tumor and hematoma. To the best of our knowledge, this is the first reported case of HCC metastasized to the cauda equina with tumor bleeding causing cauda equina syndrome.

## 1. Introduction

The majority of *cauda equina* tumors are primary tumors of glial or nerve sheath origin. Indeed, metastases from outside of the central nervous system in this region are very rare [4]. The symptoms of cauda equina lesions are known to be nonspecific. Lower back pain is the most common symptom, followed by sciatic pain, sensory disturbance, motor weakness, and bladder dysfunction [2, 3]. Up to now, only 19 cases, including this case, have been reported in the literature [6]. Herein, we describe the case of a solitary metastatic HCC to the cauda equina with tumor bleeding. To the best of our knowledge, this is the first reported case of an extramedullary-intradural spinal metastasis of hepatocellular carcinoma causing cauda equina syndrome.

## 2. Case report

A 57-year-old man presented with a 3-week history of worsening lower back pain radiating to the posterior aspect of the bilateral legs. As well, he had an acute onset of bilateral lower leg weakness for 2 days. The patient’s medical history was significant because he had undergone a liver echo guide biopsy that revealed cholangiocarcinoma in August of 2012. Furthermore, he had hepatitis B and liver cirrhosis for many years. During the physical examination, it was noted that there was a significant reduction in the range of motion in the lumbar area due to severe back pain. The pain was worsened by coughing and sneezing. The patient had poor lower muscle strength (Muscle Power: 2/2) and a decreasing sensation to pinpricks over the right thigh (L3). Both plantar responses yielded no response. Patella and Achilles tendon reflexes were diminished in both lower extremities. In addition, he had bladder-urinary dysfunction and saddle anesthesia. Tumor marker showed AFP(EIA): 13.45 ng/mL CEA(EIA): 13.76 ng/mL, PSA(EIA): 0.195 ng/mL, CA19-9(EIA): 540.2 U/mL.

**Fig. 1:**
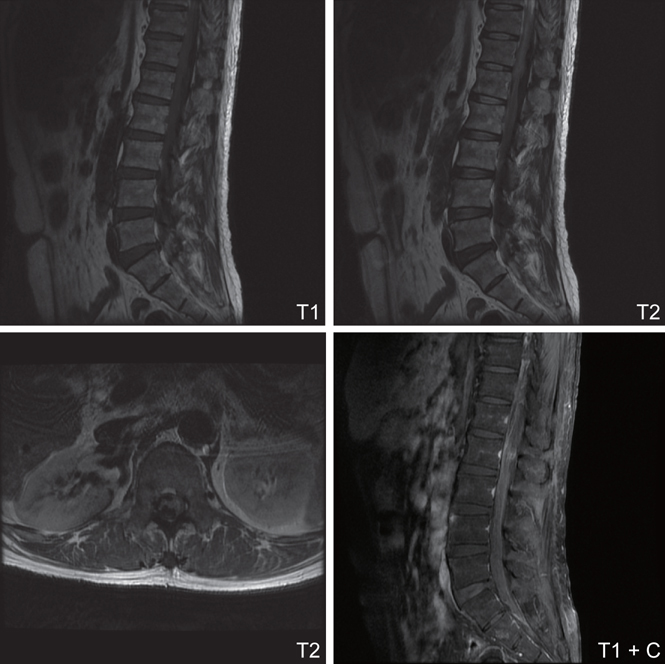
->Infiltrative hypointensity mass lesion in the dural sac from T12 to S1 level is noticed on both T1WI and T2WI. Acute hemorrhage was suspected. It compressed the spinal cord and cauda equinal nerves. Besides, there is an ill-defined intradural extramedullary mass lesion at L2 level, showing hyperintensity on T2WI and heterogeneous contrast enhancement on T1WI with contrast. Spinal metastatic tumor is highly suspected.

A lumbosacral magnetic resonance image (MRI) indicated infiltrative hypointensity in the dural sac from T12 to S1 level. compressing the spinal cord and cauda equinal nerves was noticed on both the T1WI and T2WI, leading to suspicion of an intradural hematoma. There was an ill-defined intradural extramedullary lesion at the L2 level, showing hyperintensity on T2WI and heterogeneous contrast enhancement, leading to suspicion of a metastatic tumor. (Figure 1) Magnetic resonance imaging of the brain showed multiple metastatic brain tumors with tumor bleeding. A whole body bone scan showed a T8 vertebral body metastatic lesion.

The patient underwent a laminectomy from L1 to L4 to remove the intradural spinal tumor and hematoma. When the dural sac was opened, a well-formed blood clot was noted and the tumor was found to be under the blood clot. The blood clot and the tumor together severely compressed the conus medullaris and displaced several nerve roots that ran directly into the tumor. Although attempts with delicate dissection were made to free the tumor from the roots, all of the attempts were in the end unsuccessful in saving the roots. The histopathology was diagnostic of hepatocellular carcinoma.

The patient experienced an uncomplicated postoperative course and his pain was much relieved. However, the muscle power of the bilateral lower limbs did not improve and urinary retention still persisted. He underwent radiotherapy for intraspinal lesion including T8 and L2/3 level. Because of portal vein invasion and the tumor being larger than 8 cm, the HCC was unresectable and TACE was not suitable.

## 3. Discussion

Hepatocellular carcinoma (HCC) is an aggressive tumor that frequently occurs in the setting of chronic liver disease and cirrhosis. The incidence of extrahepatic metastasis reportedly ranges from 13.5% to 36.7%, and the lungs, adrenal glands, and bone marrow are the sites where HCC frequently metastasises [4]. Metastasis of HCC to the central nervous system (CNS) is uncommon, and this relatively lower incidence of CNS metastasis may be due to the low affinity of HCC for the nervous system and the rapid disease course and short survival time of patients with HCC, which decreases the likelihood of the development of CNS metastases [5]. The primary lesions that metastasize to the cauda equina include the kidney, lung, breast, anus, uterus, ovary, prostate, and bone. We are reporting here the first case of extramedullary-intradural spinal metastasis from hepatocellular carcinoma causing cauda equina syndrome.

Several routes have been hypothesized by which malignant tumour cells might reach the cauda equina [7]: (1) haematogenously *via* the arterial route, (2) through the extra-intraspinal anastomosing venous network (Batson’s plexus) [8], (3) centripetal spread via the perineural spaces [9], (4) by direct extension of an extradural mass [10], and (5) further dissemination of CNS secondaries along the subarachnoid space as “drop metastases” [11]. In our case, the most likely route of dissemination was the latter, drop metastases, because the patient had multiple cerebral metastases. Perrin *et al*. [12] reported that 90% of intraspinal metastases are associated with metastatic brain tumors. Intraspinal metastasis results when tumor cells descend through the cerebrospinal fluid by gravity. Furthermore, occurrence of acute neurological decline after spontaneous tumor hemorrhage was also noted in our case.

Treatment options for compressive space-occupying deposits have not been clearly defined, and a nonsurgical approach is recommended by several authors. Nevertheless, surgical treatment of compressive intradural metastases of the cauda equina seems to be a feasible treatment option with low operative risk and with the potential benefit of an immediate relief of pain and improvement in motor function and thus an increase in quality of life [13].

In conclusion, intradural metastasis to *cauda equina* is a rare occurrence. Our case presented with bleeding producing further neurological deterioration. Hemorrhagic presentation has only once been previously reported [14]. Therefore, it is a new factor that should be considered in differential diagnosis when a patient with a suggestive medical history has the unique pattern of pain suggestive of an intradural tumor. Also, magnetic resonance imaging is a useful tool for detecting intraspinal metastasis.
